# Size of the Financial Incentives in Medicare’s Skilled Nursing Facility Value-Based Purchasing Program

**DOI:** 10.1001/jamanetworkopen.2025.33369

**Published:** 2025-09-24

**Authors:** Robert E. Burke, Franya Hutchins, Jonathan Heintz, Syama R. Patel, Scott Appel, Julie Norman, Atul Gupta, Liam Rose, Rachel M. Werner

**Affiliations:** 1 Division of Hospital Medicine, Perelman School of Medicine, University of Pennsylvania, Philadelphia; 2 Division of General Internal Medicine, Perelman School of Medicine, University of Pennsylvania, Philadelphia; 3 Leonard Davis Institute of Health Economics, University of Pennsylvania, Philadelphia; 4 Center for Healthcare Evaluation, Research, and Promotion, Corporal Crescenz VA Medical Center, Philadelphia, Pennsylvania; 5 Center for Healthcare Evaluation, Research, and Promotion, Pittsburgh VA Medical Center, Pittsburgh, Pennsylvania; 6 Center for Clinical Epidemiology and Biostatistics, University of Pennsylvania Perelman School of Medicine, Philadelphia; 7 Health Economics Resource Center, Palo Alto VA Medical Center, Palo Alto, California; 8 The Wharton School, University of Pennsylvania, Philadelphia; 9 Stanford Surgery Policy Improvement Research and Education Center, Stanford University, Stanford, California

## Abstract

**Importance:**

The Skilled Nursing Facility Value-Based Purchasing (SNF VBP) program is the largest test to date of the use of financial incentives to incentivize reductions in hospital readmissions from SNFs. Initial evaluations suggest the program was not successful, and a key question is to what extent the size and distribution of the financial incentives may have played a role.

**Objective:**

To measure the scale and scope of the SNF VBP financial incentives in the first 3 years of the program in which incentives were assessed (2019-2021).

**Design, Setting, and Participants:**

This is a retrospective cohort study of eligible stays, associated Medicare reimbursement amounts, financial incentive percentage assessed by SNF VBP, and proportion of financial incentive to SNF net operating revenue for each participating SNF in each year of the program. All SNFs certified by the Centers for Medicare & Medicaid Services are required to participate in the VBP program. Participants included fee-for-service Medicare beneficiaries discharged from hospital to SNF for postacute care using the same inclusion and exclusion criteria as the SNF VBP program. Data were analyzed from March 2022 to December 2024.

**Exposures:**

Financial penalties (up to 2% of all Medicare fee-for-service reimbursement for that year) or bonuses (up to 3% of all Medicare fee-for-service reimbursement for that year) for performance on risk-adjusted 30-day readmission rates.

**Main Outcomes and Measures:**

The primary outcomes were the annual amount of Medicare revenue subject to financial incentive and the financial incentive percentage for SNFs in the program, calculated by multiplying the total annual fee-for-service revenue for each SNF by the incentive multiplier published in the public SNF VBP data files.

**Results:**

The analytic sample included 5 392 281 qualifying SNF stays in 14 189 SNFs. In the first 3 years of the program, the median (IQR) reimbursement adjustment across SNFs was −$10 336 (−$27 373 to $0), or 0.15% of the median SNF net operating income. Financial incentives varied greatly from year to year for individual SNFs: only 22.3% of SNFs (3167 SNFs) remained in the same quartile in all 3 years, and a substantial minority of those that changed quartiles moved more than 1 quartile from year to year (38.3% [2890 SNFs] from 2019 to 2020, and 44.3% [3101 SNFs] from 2020 to 2021).

**Conclusions and Relevance:**

This cohort study of the SNF VBP program found that financial incentives were small, with large year-to-year variability for individual SNFs. This may have limited the program’s ability to induce meaningful investments to reduce hospital readmission.

## Key Points

**Question:**

What were the size and scope of the financial bonuses or penalties received by skilled nursing facilities (SNFs) in the first 3 years of the SNF Value-Based Purchasing (VBP) program?

**Findings:**

In this cohort study of more than 5 million qualifying SNF stays in 14 189 SNFs, the median reimbursement adjustment across all SNFs and years was −$10 336, or 0.15% of average SNF operating income. In addition, the financial incentives were highly variable from year to year for most SNFs.

**Meaning:**

These findings suggest that the small size and high variability of incentives may have contributed to the relative lack of effectiveness of the SNF VBP program.

## Teaser

This cohort study examines the scale and scope of the Centers for Medicare & Medicaid Services Skilled Nursing Facility (SNF) Value-Based Purchasing program’s financial incentives in the first 3 years of the program in which incentives were assessed.

## Introduction

The Skilled Nursing Facility Value-Based Purchasing (SNF VBP) program seeks to reduce all-cause 30-day readmissions from SNF for traditional Medicare beneficiaries recently discharged from the hospital. In prior work,^1^ we found no change in 30-day readmission rates (or other beneficiary outcome measures, such as mortality rates or community discharge rates) after SNF VBP was implemented, using data through 2021.

Under SNF VBP, most SNFs receive a financial bonus or penalty up to 2% of their total traditional Medicare revenues each year, on the basis of their performance on 30-day readmission rates compared with other SNFs, or their own improvement in readmission rates over time. The program’s baseline year was 2015, and the first financial adjustments were distributed in 2019. The program posits that financial incentives are an effective lever to drive a change in outcomes. However, this may be true only if the incentives are large enough to make a meaningful difference in a SNF’s operating budget. Prior research has demonstrated little change in quality in response to the SNF VBP program.^1^ Thus, a key unanswered question is how large and how variable the financial incentives are for individual SNFs.

Prior research on the SNF VBP program has focused on year 1 of financial incentives. Those studies demonstrated that 72% of SNFs received a financial penalty,^2^ and that the odds of receiving a penalty were associated with lower previous profit margin,^3^ higher previous readmission rates,^4^ and characteristics of the SNF itself and its underlying patient population.^2,5,6^ One study^6^ found that fewer SNFs (65%) received penalties in year 2 of the program. Another study^3^ estimated that the average financial bonus in year 1 was approximately $22 000, but used Cost Report data, which do not directly measure the financial incentives for this program. To our knowledge, no prior study has directly measured the monetary value of the incentives that SNFs received through the SNF VBP program or examined whether the size of these incentives change over time. Consistent penalties could give facilities clear incentive to improve, and consistent bonuses could give facilities resources to maintain quality, but inconsistent and/or limited pecuniary incentives may not be sufficient to motivate changes. Thus, we sought to quantify the size of SNF VBP financial incentives and changes in the financial incentives over time to help shed light on why the program may not have achieved its primary aim.

## Methods

This cohort study was reviewed and considered exempt from review and the need for informed consent by the University of Pennsylvania institutional review board, because no individual patient data were used, in accordance with 45 CFR §46. We followed the Strengthening the Reporting of Observational Studies in Epidemiology (STROBE) reporting guidelines for cohort studies.^7^ To calculate each SNF’s annual Medicare fee-for-service revenue, we used 100% MedPAR files to identify all eligible SNF stays, and the Medicare Master Beneficiary Summary File to identify fee-for-service enrollment by month from October 2018 through September 2021. Although the SNF VBP program evaluates readmissions for a subset of visits following eligibility criteria,^8^ reimbursement adjustments are applied to revenue from all fee-for-service visits. All SNFs certified by the Centers for Medicare & Medicaid Services (CMS) are required to participate in SNF VBP (see eFigure 1 in Supplement 1 for details on cohort creation).

### Statistical Analysis

Data were analyzed from March 2022 to December 2024. We calculated Medicare’s total annual fee-for-service revenue for these SNF stays using the total payment and pass-through amount (which includes capital costs that are part of SNF payments by Medicare) in MedPAR. To calculate each SNF’s incentive payment (bonus or penalty), we then multiplied the total annual fee-for-service revenue for each SNF by the incentive multiplier published in the public SNF VBP data files from CMS.^9^

We calculated the overall distribution of financial incentives both in terms of total dollars and as a percentage of net operating revenue across all SNFs in each year (2019-2021). To calculate net operating revenue, we used CMS’s SNF Cost Reports files. Specifically, following prior studies, we calculated total net revenue summing net inpatient revenues (workbook G3, line 3) plus total other income (workbook G3, line 25).^10^ We then divided the size of the financial incentive by the size of the net operating revenue of each SNF for each year.

Then, we displayed variability in financial incentives SNFs experienced year-to-year in 2 ways. First, we created a caterpillar plot to portray both the distribution of the mean financial incentive across facilities, as well as within-facility variability in financial incentive, adding error bars to demonstrate the range of minimum to maximum financial incentive around the mean value for each SNF from 2019 to 2021. Then, we divided SNFs into quartiles by the size of each SNF’s reimbursement adjustment in 2019 and examined how many remained in the same quartile vs changed quartiles each year using an alluvial plot.

## Results

**Figure 1.**
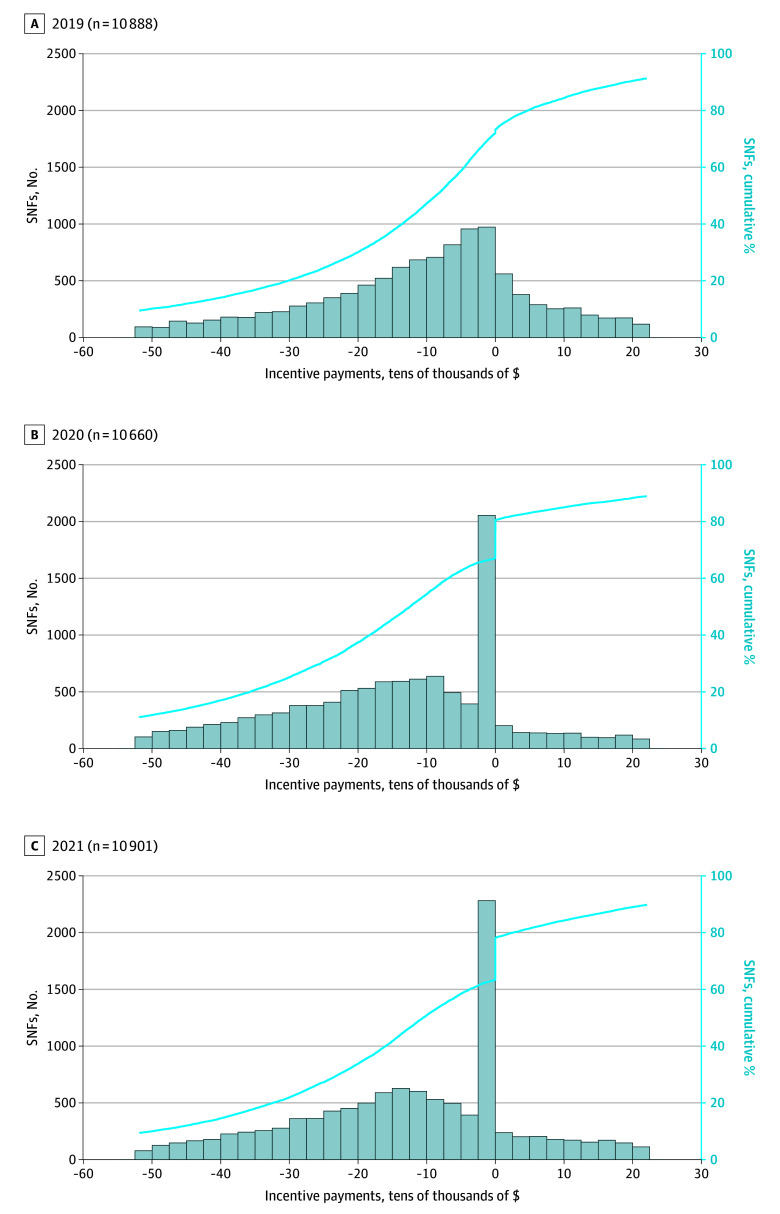
Distribution of Incentive Payment Amounts by Skilled Nursing Facility (SNF) and Fiscal Year, 2019 to 2021 The figure represents a total of 12 561 unique SNFs accounting for 32 449 SNF-years, for fiscal years 2019 (A), 2020 (B), and 2021 (C). The x-axis is the amount of incentive payment received by individual SNFs, with values less than zero connoting a penalty and greater than zero connoting a bonus payment. Values on the x-axis should be multiplied by $10 000 to get the total incentive amount. The left-sided y-axis represents the count of SNFs receiving that level of incentive payment displayed as vertical bars. The right-sided y-axis is a cumulative distribution function (displayed by a solid line) demonstrating the proportion of SNFs that have received up to a particular incentive payment. For example, in 2019, 70% of SNFs had received a negative incentive payment (0 or below). The distribution is truncated at extreme negative and positive values to display the 10th through 90th percentile.

Our analytic sample included 5 392 281 qualifying SNF stays. These stays occurred in 14 189 SNFs (not all SNFs contributed data to all 3 years, because of SNF closures and openings over the time period). Total financial incentive across all SNFs exceeded $571 million across the first 3 years, and two-thirds of SNFs received a penalty (67.3% [27 557 SNF-years], including 28.4% [11 634 SNF-years] receiving the maximum penalty). However, the impact at an individual SNF level was relatively small. The median (IQR) overall reimbursement adjustment was −$10 336 (−$27 373 to $0), or 0.15% of the average SNF net operating income (Table). Even if every SNF had received the maximum 2% penalty, the median (IQR) reimbursement adjustment would be $23 950 ($12 729 to $43 373). The distribution of reimbursement adjustments across SNFs remained similar across the 3 years, with the exception of a growing number of facilities experiencing the smallest amount of financial penalty (just below zero) in 2020 and 2021. Figure 1 shows the incentive payment amount distribution. eFigure 2 in Supplement 1 shows the net operating income distribution.

**Table.** Financial Incentives Assessed in the First 3 Years of the CMS SNF Value-Based Purchasing Program, 2019-2021

Variable	All years (N = 14 189 unique SNFs; N = 40 944 SNF-years)	FY 2019 (n = 13 463 SNFs)	FY 2020 (n = 13 746 SNFs)	FY 2021 (n = 13 733 SNFs)
Performance (measurement) period	Jan 1, 2017, to Sep 30, 2019	Jan 1, 2017, to Dec 31, 2017	Oct 1, 2017, to Sep 30, 2018	Oct 1, 2018, to Sep 30, 2019
Range of incentive multipliers	0.9802 to 1.0312	0.9802 to 1.0165	0.9803 to 1.0312	0.9803 to 1.0213
SNFs penalized, No. (%)	27 557 (67.3) SNF-years	9689 (72.0)	9198 (66.9)	8670 (63.1)
SNFs at maximum penalty (defined as incentive multiplier <0.9805), No. (%)	11 634 (28.4) SNF-years	3165 (23.5)	4735 (34.5)	3734 (27.2)
Reimbursement adjustment as amount, median (IQR), $	−10 336 (−27 373 to 0)	−8522 (−24 278 to 775)	−12 400 (−30 103 to 0)	−10 392 (−27 150 to 0)
Reimbursement adjustment, median (IQR), % of total SNF net operating income	−0.15 (−0.34 to 0.00)	−0.17 (−0.55 to 0.02)	−0.15 (−0.32 to 0.00)	−0.12 (−0.30 to 0.00)
CMS reported total penalties, $	−571 063 835	−196 084 538	−193 660 594	−181 318 703

Abbreviations: FY, fiscal year; CMS, Centers for Medicare & Medicaid Services; SNF, skilled nursing facility.

**Figure 2.**
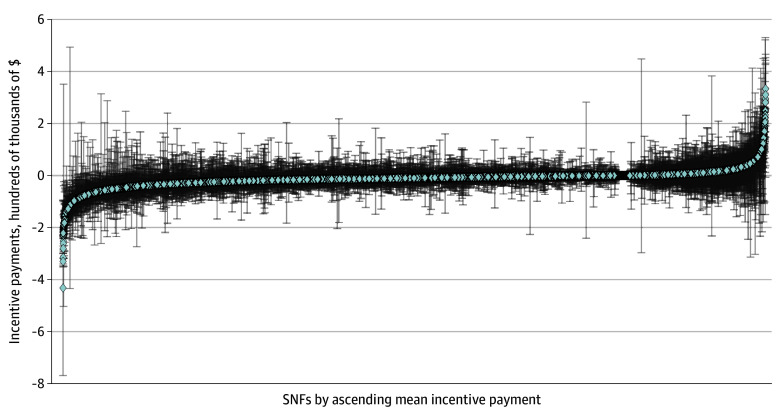
Distribution of Mean and Range of Incentive Payment Amounts by Skilled Nursing Facilities (SNFs), 2019 to 2021 The figure represents 13 808 unique SNFs with at least 2 years of data. Each data point represents the mean incentive payment (multiplied by $100 000) of one of the SNFs in our sample across all 3 years of SNF value-based purchasing measured (2019-2021). Error bars represent the maximum and minimum financial incentives received by that SNF across any of the 3 years of the program. In the caterpillar plot, SNFs are organized from those with the lowest mean (largest financial penalty) on the left to the highest mean (largest financial bonus) on the right.

However, individual SNFs experienced substantial variability in reimbursement adjustments from year to year. Figure 2 shows incentive payment amount. eFigure 3 in Supplement 1 shows net operating income. Only 22.3% of SNFs (3167 SNFs) remained in the same quartile in all 3 years (28.3% [896 SNFs] of those in the highest quartile with highest incentive payment multiplier, 14.8% [470 SNFs] in the second quartile, 33.4% [1059 SNFs] in the third quartile, and 23.4% [742 SNFs] of those in the lowest quartile with the lowest incentive multiplier). A substantial minority of those that changed quartiles moved more than 1 quartile from year to year (38.3% [2890 SNFs] from 2019 to 2020, and 44.3% [3101 SNFs] from 2020 to 2021) (eFigure 4 in Supplement 1).

## Discussion

In this cohort study, we found that the size of the financial incentives at the SNF level are relatively small in terms of dollars and as a proportion of net operating income, and that most SNFs experienced substantial variability from year to year in their incentive payments. These 2 factors may have contributed to the relative lack of effectiveness of the SNF VBP program. First, if the level of the penalty is not sufficient to hire additional staff, purchase equipment (such as an x-ray machine or laboratory testing), or invest in new care processes, then SNFs will not be able to improve their ability to manage changes in patient condition. Although the Hospital Readmission Reduction Program (HRRP) had a similar proportion of penalty to net operating income, the financial incentive was much larger: the median financial incentive was more than $200 000.^11,12^ This larger absolute size of the financial incentive could have helped hospitals invest in additional staff or resources, although it is not clear why the HRRP was relatively more successful than SNF VBP in reducing hospital readmissions.^12^ Although the small size of the financial incentives is likely much more important in explaining the lack of effectiveness of the program than the year-to-year variability in incentives, even SNFs whose incentives were relatively large in absolute terms (large enough to invest in new resources to prevent readmissions) often could not count on a consistent funding stream to support these efforts.

On reviewing these results, many might wonder why larger financial incentives were not used in the program. The evidence regarding whether higher-intensity financial incentives are more likely to result in success is mixed, with some reports indicating they are more likely to improve outcomes,^13^ but prior VBPs in nursing homes, even with larger potential upside and no downside risk, were similarly unsuccessful.^14^ Although estimates of SNF profitability vary greatly, there may have been justifiable concerns about consistent penalties leading to SNF closures and decreased access for older adults. Second, the penalty size as a proportion of net operating income mirrors that of HRRP, which was successful in reducing readmission rates.^15^ More broadly, our findings suggest that renewed focus on what is required for SNFs to make structural and process changes to improve outcomes is needed.

### Limitations

Strengths of the analysis included robust ascertainment of SNF VBP-eligible stays and Medicare payments. However, the SNF Cost Report data we used to calculate net operating income may inaccurately estimate SNF revenue and profit, since SNFs may tunnel profits to other entities with the same owner.^16,17^

## Conclusions

In this cohort study of more than 5 million qualifying SNF stays in 14 189 SNFs in the first 3 years of the SNF VBP program where financial incentives were assessed, we found financial incentives were small and highly variable, on average, at an individual SNF level. The SNF VBP program will incorporate additional outcome measures in coming years, which may provide an opportunity to understand whether smaller incentives are effective for different outcome measures.^18^ Our results suggest varying the size of the financial risk and reward may also be worth testing to improve the impact of the SNF VBP program.

## Notes

Supplement 1. **eFigure 1.** Visit and Skilled Nursing Facility Flow-Chart, Fiscal Years 2019-2021 **eFigure 2.** Distribution of Incentive Payment as a Percentage of SNF Net Operating Income by SNF and Fiscal Year, 2019-2021 **eFigure 3.** Distribution of Mean and Range of Incentive Payments as a Percentage of Net Operating Income by SNF, 2019-2021 **eFigure 4.** Change in Quartile of Incentive Payment as Percent of Net Operating Income by SNF and Fiscal Year, 2019-2021

Supplement 2.
Data Sharing Statement
