# Supplementary material for: Size of the Financial Incentives in Medicare’s Skilled Nursing Facility Value-Based Purchasing Program
Source: JAMA Netw Open. 2025 Sep 24;8(9):e2533369. doi: 10.1001/jamanetworkopen.2025.33369 (PMC12461404; doi:10.1001/jamanetworkopen.2025.33369)
Supplement: Supplement 1. — eFigure 1. Visit and Skilled Nursing Facility Flow-Chart, Fiscal Years 2019-2021 eFigure 2. Distribution of Incentive Payment as a Percentage of SNF Net Operating Income by SNF and Fiscal Year, 2019-2021 eFigure 3. Distribution of Mean and Range of Incentive Payments as a Percentage of Net Operating Income by SNF, 2019-2021 eFigure 4. Change in Quartile of Incentive Payment as Percent of Net Operating Income by SNF and Fiscal Year, 2019-2021 [file jamanetwopen-e2533369-s001.pdf]

Supplemental Online Content

Burke RE, Hutchins F, Heintz J, et al. Size of the financial incentives in Medicare's Skilled Nursing Facility Value-Based Purchasing Program. *JAMA Netw Open*. 2025;8(9):e2533369. doi:10.1001/jamanetworkopen.2025.33369

eFigure 1. Visit and Skilled Nursing Facility Flow-Chart, Fiscal Years 2019-2021

eFigure 2. Distribution of Incentive Payment as a Percentage of SNF Net Operating Income by SNF and Fiscal Year, 2019-2021

eFigure 3. Distribution of Mean and Range of Incentive Payments as a Percentage of Net Operating Income by SNF, 2019-2021

eFigure 4. Change in Quartile of Incentive Payment as Percent of Net Operating Income by SNF and Fiscal Year, 2019-2021

This supplemental material has been provided by the authors to give readers additional information about their work.

© 2025 Burke RE et al. *JAMA Network Open*.

eFigure 1: Visit and Skilled Nursing Facility Flow-Chart, Fiscal Years 2019-2021

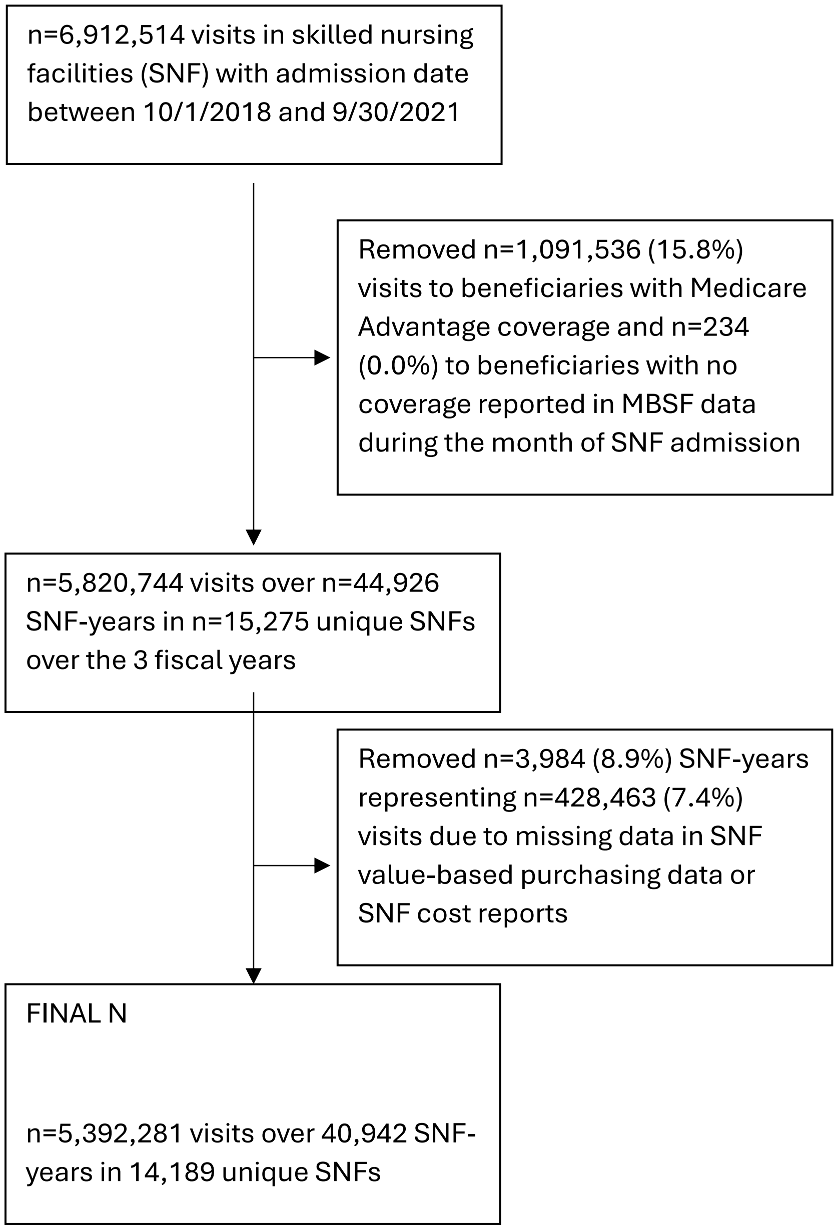

© 2025 Burke RE et al. *JAMA Network Open*.

eFigure 2: Distribution of Incentive Payment as a Percentage of SNF Net Operating Income by SNF and Fiscal Year, 2019-2021

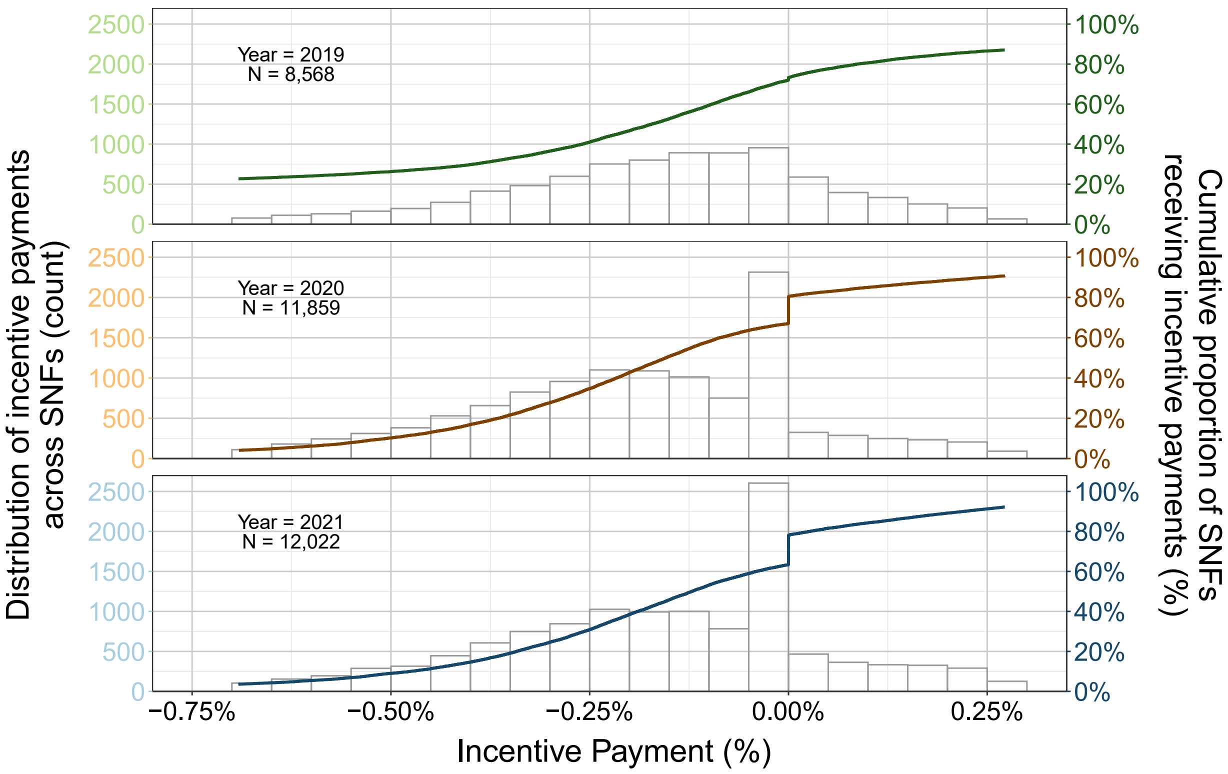

Legend: The figure represents a total of n= 12,561 unique skilled nursing facilities (SNF) accounting for 32,449 SNF-years. The annual number of SNF's presented is shown in each panel. Incentive payment percent values are calculated out of the absolute value of each SNF's net operating income per fiscal year. Negative values indicate penalty. Overlayed lines are the within-year cumulative percent of SNFs. Data are truncated to show the 10th through 90th percentiles of percent incentive payment.

© 2025 Burke RE et al. *JAMA Network Open*.

eFigure 3: Distribution of Mean and Range of Incentive Payments as a Percentage of Net Operating Income by SNF, 2019-2021

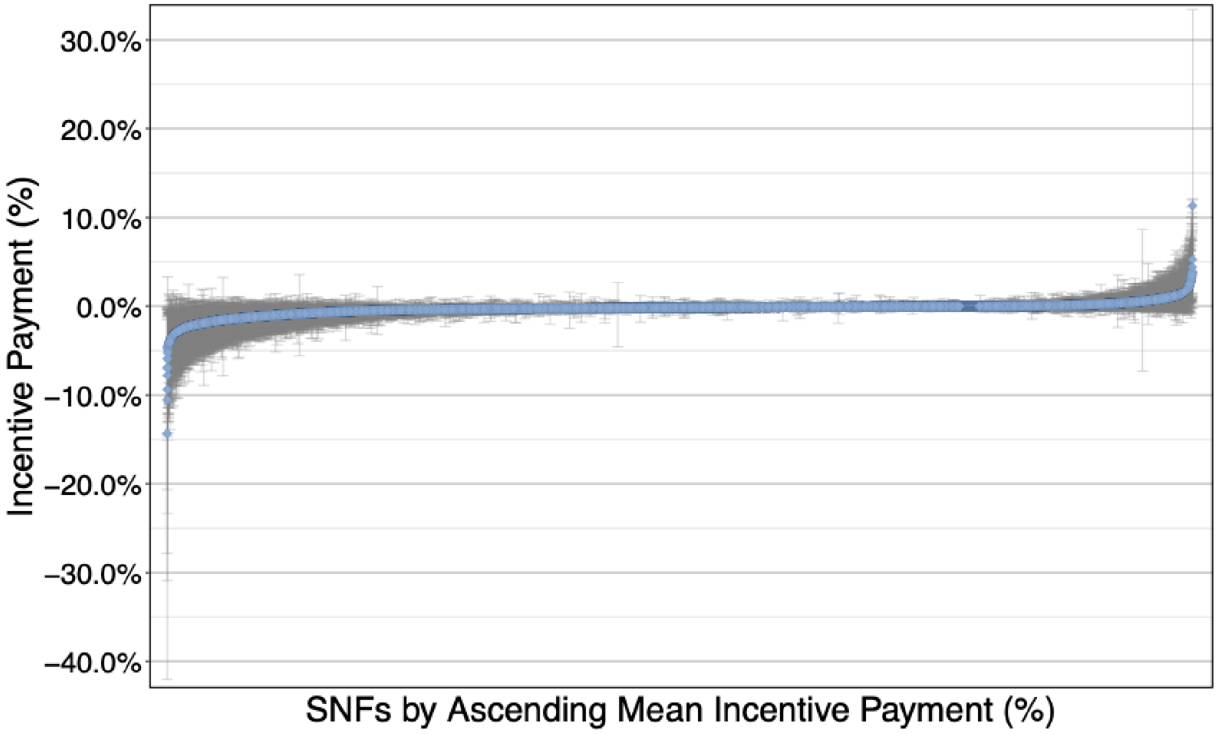

Legend: The figure represents n=13,808 unique skilled nursing facilities with at least 2 years of data. Each blue dot represents the mean incentive payment as a percentage of net operating income of one of the SNFs in our sample across all three years of SNF VBP measured (2019-2021). The "whiskers" extending vertically in each direction from the mean represents the maximum and minimum financial incentives as a percentage of net operating income received by that SNF across any of the three years of the program. In the caterpillar plot, SNFs are organized from those with the lowest proportion (largest financial penalty) on the left to the highest proportion (largest financial bonus) on the right. Incentive payment percent values are calculated out of the absolute value of each SNF's net operating income per fiscal year.

© 2025 Burke RE et al. *JAMA Network Open*.

eFigure 4: Change in Quartile of Incentive Payment as Percent of Net Operating Income by SNF and Fiscal Year, 2019-2021

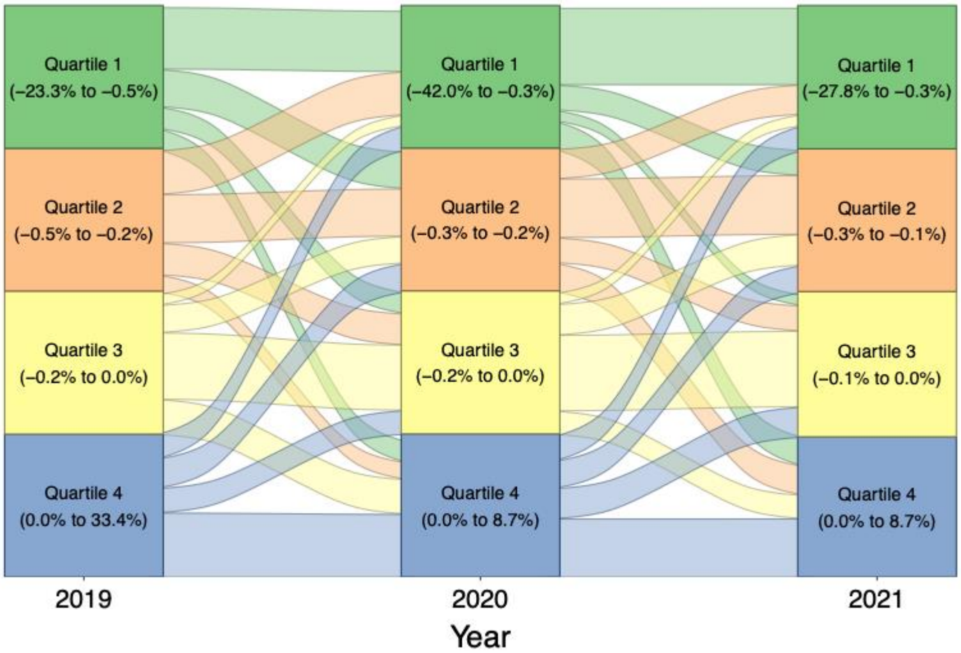

The figure represents n= 14,189 unique SNFs accounting for a total of n= 40,942 SNF-years. Incentive payment percent values are calculated out of the absolute value of each SNF's net operating income per fiscal year. Negative values indicate penalty.

© 2025 Burke RE et al. *JAMA Network Open*.
