# Supplementary material for: Size of the Financial Incentives in Medicare’s Skilled Nursing Facility Value-Based Purchasing Program
Source: JAMA Netw Open. 2025 Sep 24;8(9):e2533369. doi: 10.1001/jamanetworkopen.2025.33369 (PMC12461404; doi:10.1001/jamanetworkopen.2025.33369)
Supplement: Supplement 2. — Data Sharing Statement [file jamanetwopen-e2533369-s002.pdf]

# Data Sharing Statement

Burke. Size of the Financial Incentives in Medicare's Skilled Nursing Facility Value-Based Purchasing Program. *JAMA Netw Open*. Published September 24, 2025. doi:10.1001/jamanetworkopen.2025.33369

**Data**
**Data available:** No
